# Integrating Magnetic Resonance Chemical Shift Imaging for Localized Prostate Cancer Risk Stratification on the Basis of the Impact of Periprostatic Brown Adipocytes Within Tumor Microenvironment

**DOI:** 10.1245/s10434-025-17512-5

**Published:** 2025-05-28

**Authors:** Chih-Yu Shen, Jhih-Kai Pan, Wen-Der Lin, Hui-Chuan Cheng, Che-Yuan Hu, Kuan-Yu Wu, Yung-Ming Kuo, Gia-Shing Shieh, Pei-Jung Lu

**Affiliations:** 1 Institute of Clinical Medicine, College of Medicine, National Cheng Kung University, Tainan, Taiwan; 2 Department of Urology, Tainan Hospital, Ministry of Health and Welfare, Tainan, Taiwan; 3 Department of Urology, National Cheng Kung University Hospital, College of Medicine, National Cheng Kung University, Tainan, Taiwan; 4 Department of Electronic Engineering, College of Electrical and Computer of Engineering, National Formosa University, Tainan, Taiwan

**Keywords:** Prostate cancer, Magnetic resonance imaging, Brown adipocyte, Risk stratification, Periprostatic adipose tissue

## Abstract

**Background:**

We sought to characterize periprostatic adipose tissue (PPAT) with magnetic resonance imaging (MRI) featuring water-to-oil ratio (*R*^WO^) to detect brown adipocyte (BAT).

**Patients and Methods:**

Between November 2021 and September 2023, 21 localized patients with prostate cancer were studied, categorized as low (*n* = 4), intermediate (*n* = 4), and high risk (*n* = 13). We utilized MRI to analyze the water-only signal and fat-only signal. *R*^WO^ was used to predict the risk stratification. Tissue samples, including periprostate fat, were collected during surgery, processed, and stained with hemotoxylin and eosin (H&E) for microscopic analysis. Periprostate adipose cell supernatants were used to treat cancer cells (PC3 and LNCaP) in vitro.

**Results:**

We found significantly higher periprostatic adipose tissue *R*^WO^ in the high and intermediate risk groups compared with the low risk group (52.12 versus 30.48; *p* < 0.0001). The receiver operating characteristic curve for distinguishing advanced tumors using PPAT *R*^WO^ in MRI imaging yielded an area under the curve of 0.64, which increased to 0.90 after incorporating initial PSA. Immunofluorescence, H&E staining, and immunohistochemistry revealed the presence of brown adipocytes, marked by uncoupling protein 1 expression, in periprostate tumor fat. Results indicate that BAT-related adipokines promote epithelial-mesenchymal transition and invasiveness in human prostate cancer cells.

**Conclusions:**

In the study, the chemical shift image of MRI in 21 localized patients with prostate cancer revealed higher periprostatic adipose tissue water-to-oil ratio among patients with high-risk prostate cancer. Adipokines within the tumor-microenvironment attribute to the cancer aggressiveness, and targeting the fat fraction signal in MRI could improve the current risk stratification strategy.

**Supplementary Information:**

The online version contains supplementary material available at 10.1245/s10434-025-17512-5.

Prostate cancer is a common malignancy among men globally, representing one in five new cancer diagnoses in this group.^1^ Recent studies have emphasized the crucial role of the tumor microenvironment in the development and progression of this disease. Adipocytes are integral to the tumor microenvironment and significantly influence prostate cancer biology. Magnetic resonance imaging (MRI) provides noninvasive insights into the role of adipocytes in prostate cancer. MRI is used to evaluate body fat owing to its detailed soft-tissue characterization, superior spatial resolution, and absence of ionizing radiation exposure for patients. Previous results suggest that increased periprostatic fat volume, normalized to prostate size, may be associated with shorter progression-free survival in men with prostate cancer undergoing active surveillance.^2^ Besides periprostatic fat volume, the composition of periprostatic fat, particularly near the tumor region, might influence the tumor microenvironment and could potentially be detected with MRI imaging.

Brown adipose tissue (BAT) is known for thermogenesis, consuming fat in response to stimuli such as low temperatures, at the molecular level, is carried out by the mitochondrial uncoupling protein 1 (UCP-1/thermogenin).^3,4^ It has been thought that adult humans have small BAT depots confined to specific regions, including the cervical, supraclavicular, and axillary areas, as well as perivascular regions and near the kidneys. Nevertheless recently, Leitner and coworkers have accurately mapped the human BAT depots, using positron emission tomography/computed tomography, showing that previous studies have underestimated the presence of this tissue in adults.^5^ The MR imaging has been shown to have the potential to distinguish BAT and WAT on the basis of two independent physiologic factors: BAT has a higher water-to-fat ratio than WAT, and BAT has a high density of mitochondria and blood vessels.^6^ A reliable MR protocol to measure BAT volume and distribution in living adult humans is still lacking. In the current study, we use sequences available in most clinical MR scanners to access BAT location and function.

Periprostatic adipose tissue (PPAT) surrounds the prostate gland and is thought to contribute to the progression of prostate cancer. Alix Fontaine et al. demonstrate that the coculture of prostate cancer (PCa) cell lines with adipocytes decreased autophagy activity and increased lipid droplets flux in PC3 cells. In locally advanced PCa, markers of autophagy and lipid droplets staining were increased in extraprostatic areas where cancer cells were in contact with PPAT,^7^ which we hypothesis could be detected via MRI. Zhang et al. showed a correlation between the aggressiveness of prostate cancer and periprostatic fat area seen on a single transverse MRI section at the level of the femoral head and greater trochanter.^8^

Understanding the relationship between prostate cancer and adipocytes opens potential avenues for targeted therapies. On the basis of the presence of BAT in PPAT, we hypothesize that multiparametric magnetic resonance imaging (mpMRI) incorporating chemical shift imaging detecting BAT on the basis of a higher water-to-fat ratio, when combined with serum prostate-specific antigen (PSA), could serve as a promising noninvasive predictive tool in prostate cancer risk stratification.

## Patients and Methods

### Patients

**Fig. 1:**
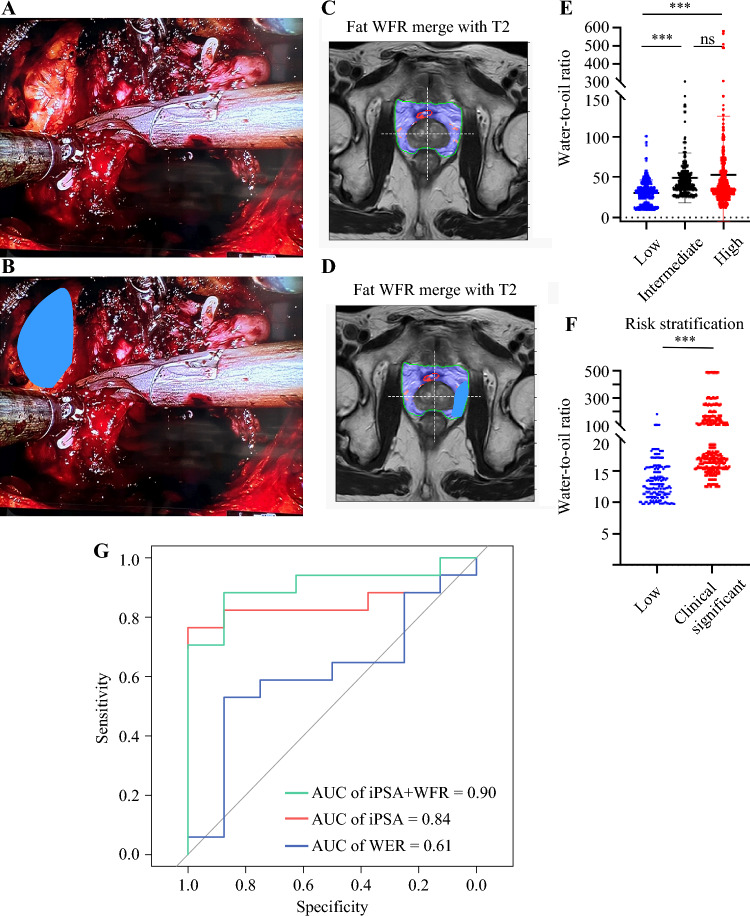
**A**, **B** Collection method for preprostate fat during the operation; after developing the space of Retzius and anterior prostate dissection, direct visualization of the anterior prostate and periprostatic fat was dissected; **C**, **D** chemical shift MRI imaging of water-to-oil fraction incorporated into T2 series showing the orientation of periprostate tumor fat collected during the operation; **E**, **F** significantly higher water-to-oil ratio signal of periprostate tumor fat was shown in high-risk patient compared with that in low-risk patients; **G** receiver operating characteristic curve showing area under curve of 0.90 in determining high risk patient from iPSA and water-fat-ratio imaging

A prospective cohort study on patients with prostate cancer, with institutional review board approval (no. A-ER-110-223) and waived informed consent requirements, involved MRI imaging and clinical assessments. Inclusion criteria included men with an abnormal digital rectal examination and elevated PSA who underwent a transrectal ultrasound (TRUS) biopsy or MRI/ultrasound fusion biopsy. Patient demographics, PSA levels, and metastasis data were recorded. Treatments included prostatectomy with or without androgen-deprivation therapy. Tissue samples, including periprostate and subcutaneous fat, were collected during surgery, processed, and stained with hemotoxylin and eosin (H&E) for microscopic analysis (Fig. 1A,B).

### Imaging Protocols

We used a noncommercial version of the mDIXON (Philips Healthcare) chemical-shift water-fat multiecho pulse sequence, generating fat-signal fraction maps reflecting proton density ratios between fat and the sum of fat and water. MRI scans were performed on 1.5-T Siemens MR units. Each scan was reviewed by an attending radiologist. Periprostatic adipose tissue (PPAT) volume, defined as the fat surrounding the prostate and anterior to the rectum, was outlined including the first visible fascial boundary adjacent to the levator muscles laterally, Denonvilliers fascia posteriorly, and the pubic symphysis anteriorly.^9^ We measured PPAT volume on multiple transverse T2-weighted planes from multiple sequential slices starting from apex to base of prostates (Fig. 1C,D).

In the study of brown fat in periprostatic fat, MRI was utilized to analyze the water-only signal (WOS) and fat-only signal (FOS) (Supplementary Fig. 1A). A two-dimensional slice was selected from the three-dimensional MRI data, and the slice’s WOS and FOS, represented as Water Only Matrix (WOM) and Fat Only Matrix (FOM), were computed to identify brown fat. To visually display intensity variations in WOM and FOM, each element’s intensity, originally at 4096 levels, was converted to 256 levels using a specific equation Eq. X.

$${X}_{\text{new}}=\text{round}\left(\frac{ X - {X}_{\text{min}}}{{X}_{\text{max}}-{X}_{\text{min}}} \times 255\right)(\text{Eq}.\text{ X})$$

where *X* is the original data, *X*_max_ is 4095, *X*_min_ is 0, and *X*_new_ is represented as a converted data point of *X*. The corresponding images are shown as Image of WOM (IWO, Supplementary Fig. 1B) and Image of FOM (IFO, Supplementary Fig. 1C) respectively. We calculate the matrix of water-to-oil ratio (referred to as *R*^WO^) from WOM and FOM and extract the brown fat in periprostatic fat from *R*^WO^. The height and width of WOM and FOM are denoted as *H* and *W*, where WOM and FOM represent the values of the water and fat components at position (*h*, *w*) in WOM and FOM, respectively. The following equation (Eq. Y) is then used to calculate the water-to-oil ratio for each element, ultimately obtaining *R*^WO^.^6^

$${R}_{\left(h,w\right)}^{\text{WO}}=\frac{{\text{WOM}}_{\left(h,w\right)}}{{\text{FOM}}_{\left(h,w\right)}} \left(\text{Eq}.\text{ Y}\right)$$

To visualize *R*^WO^ using image, we employ the converting method, similar to Eq. X, to transform the value of each element in RWO to the range of 0 to 255. The resulting image of RWO is denoted as IRWO (Supplementary Fig. 1D). To obtain the brown fat in the periprostatic fat, the first step is to set the elements’ value of the non-periprostatic-fat area of *R*^WO^ to zero. The result matrix is represented by PR^WO^, which represents the periprostatic fat of *R*^WO^. Then, all elements in PR^WO^ are sorted from large to small by value. Finally, on the basis of the sorting, the values of the first 1% of elements are retained on the basis of on the scarce amount of the brown fat, and the values of other elements are set to zero. The resulting matrix is represented by Brown Fat of PR^WO^ (BPR^WO^). Here, the converting method, similar to Eq. X, is used to obtain the image of BPR^WO^, denoted as IBPR^WO^ (Supplementary Fig. 1E). In IBPR^WO^, the periprostatic fat is in the region inside the green contour and outside the red contour (Supplementary Fig. 1F).

### Primary Adipocyte Cell Culture

The PPAT collected during radical prostatectomy was placed in a sterile 10-cm dish, bathed in Hank’s balanced salt solution (HBSS), and cut into approximately 1-cm^2^ pieces using sterile scissors and thumb forceps, which was then placed approximately into centrifuge tubes. Following incubation with 25 ml of sterile enzyme solution for 1 h at 37 °C, the tissue was filtered in through a sterile 1000-ml plastic mesh in a sterile funnel into a clean sterile 50-ml centrifuge tube. After spinning the filtrate in a centrifuge for 10 min at 1000 rpm, the underlying pellet containing preadipocytes, fibroblasts, and erythrocytes were removed. After the last centrifugation step, the fatty layer containing the mature adipocytes was transferred to 12.5 cm^2^ cell culture flasks, which were then filled completely with Dulbecco’s modified Eagle’s medium (DMEM) and 10% fetal bovine serum (FBS), then inverted so that the bottom of the flask was on top. The flasks were incubated at 37 °C in a 5% CO_2_ incubator for cell attachment. After sufficient attachment of the cells, usually 5–7 days, we removed the medium, replaced with 5 ml fresh medium, and re-inverted the flasks.^10,11^

### Quantitative Real-Time PCR (qPCR)

BAT-selective markers included the UCP-1, TBX-1, LHX8, and two brown-versus-white markers,^12,13^ also clustered with the BAT-selective group. Wu et al. found that TBX1 and TMEM26, two markers recently defined as brite in a screening of immortalized brite and brown cell lines, also ended up in the BAT-selective group.^14^ HOXC8 and HOXC9 are more highly expressed in WAT and brite adipocytes compared with BAT.^12,15,16^. We use UCP1, LHX8, and TBX1 for detecting BAT and HOXC8, HOXC9, and leptin for WAT. Total RNA isolation and reverse transcription was conducted. The results are normalized to those of the housekeeping gene alpha-tubulin.

### RNA Purification

RNA was extracted from cultured prostate cancer cells using the Tri-Reagent method. Briefly, cells were harvested at 80–90% confluency and lysed directly in the culture dish using Tri-Reagent according to the manufacturer’s instructions. For tissue samples, approximately 30 mg of tissue was homogenized in Tri-Reagent using a mechanical tissue homogenizer. Following lysis, the samples were incubated at room temperature for 5 min to ensure complete denaturation of cellular proteins and inactivation of RNases.^17^ Chloroform was then added to the lysate, and vigorous shaking followed by centrifugation was performed to achieve phase separation. The upper aqueous phase, containing RNA, was carefully collected and mixed with isopropanol for RNA precipitation. After incubation and centrifugation, the RNA pellet was washed with 70% ethanol, air-dried, and reconstituted in diethyl pyrocarbonate (DEPC)-treated water. The purity and concentration of the isolated RNA were assessed using a NanoDrop spectrophotometer (Thermo Fisher Scientific, Waltham, MA, USA), and the integrity was confirmed by gel electrophoresis. The extracted RNA was subsequently stored at −80 °C until further analysis. All steps were conducted under RNase-free conditions to ensure the integrity of the RNA samples.^18^

### Invasion and Migration Assay

Periprostate adipose cell supernatants are used to treat cancer cells PC3 and LNCaP (ATCC) in vitro. Migration and invasion assays involve Transwell chambers with 8-mm membrane filters.^19^ Cells, trypsinized and serum-starved for 24 h, are resuspended in serum-free medium; 1.5 × 10^5^ cells are placed in the upper chamber, and the lower chamber contained medium with 10% FBS. After 48 h, cells that migrated through the membrane are fixed, stained, and counted under a microscope in four random fields.^18^

### Coculture

PC-3 and LNCaP cell lines are cultured in Roswell Park Memorial Institute (RPMI)-1640, supplemented with 10% fetal bovine serum and antibiotics. For coculture experiments, nonfluorescent human PPAT is seeded first; after they adhere, cancer cells (1.0 × 10^5^) are seeded on topper well of a 96-well plate. Analysis is performed after 24 h of coculture. Cancer cell numbers in culture are determined by total nucleated cell counts.

### Western Blot

The protein samples are boiled to denature and transferred to a membrane following electrophoretic separation. The proteins are blocked with milk to prevent nonspecific antibody binding, and then they are stained with antibodies specific to the target protein. The membrane will be stained with a secondary antibody that recognizes the first antibody staining, which can then be used for detection.

### Statistics

In our study, normally distributed continuous variables are expressed as mean values with standard deviations and are compared using unpaired two-tailed Student’s *t*-tests. Nonnormally distributed continuous variables are expressed as median and interquartile ranges and are compared using the Mann–Whitney test. Categorical variables are compared using the chi-squared test or Fisher’s exact test as appropriate. The Spearman correlation coefficient is used to evaluate the relationship between PPAT amount and clinical parameters. The statistical analysis is performed using Prism5 and SPSS17 software (New York, NY, USA). Cox proportional hazards regression models with univariate and multivariate analyses are summarized with hazard ratios and 95% confidence intervals. For all tests, *p* < 0.05 defines statistical significance (*p < 0.05, **p < 0.01, ***p < 0.001). ROC curve analysis was performed to assess the diagnostic accuracy of the classification system.

## Results

A schematic diagram of accessing the periprostate fat during operation is shown in Fig. 1A and B. MRI cognitive adipose tissue biopsy near prostate and distant from tumor was performed (Fig. 1C,D). To investigate the relationship between chemical shift MRI and prostate cancer risk stratification, this study, conducted from November 2021 to September 2023, enrolled 21 patients (as detailed in Table 1). In our study, the mean *R*^WO^ of PPAT varied across prostate cancer risk groups, being 30.5 (standard deviation, SD 16.0) for low-risk, 49.0 (SD 30.7) for intermediate-risk, and 52.9 (SD 72.5) for high-risk (Fig. 1E). Notably, the clinically significant tumors (defined as high-risk and intermediate-risk groups) showed significantly higher PPAT *R*^WO^ compared with the low-risk group (mean 52.12 versus 30.48; 95% CI: 16.32 to 26.94; *p* < 0.0001) (Fig. 1E,F). Moreover, they showed a relatively concentrated signal within the periprostate tumor region in clinically significant tumors compared with that of the low risk group (Supplementary Fig. 2). These findings suggest an increase in lipolysis within the tumor microenvironment adjacent to prostate cancer tissue.

**Table 1:** Patient characteristics and Risk stratification (N=21)

	Risk stratification according to the National Comprehensive Cancer Network (NCCN)			
Patient characteristics	Low (N=4)	Intermediate (N=4)	High (N=13)	*p* value
Age (SD)	72.5(8.7)	69.7 (6.9)	70.8 (10.4)	0.24
iPSA (ng/ml)(SD)	6.7 (2.3)	10.4(3.6)	51.9(50.6)	<.001
Gleason grade group, n (%)				<.001
1	4 (100.0)	1 (25.0)	1 (7.6)	
2	0	0	0	
3	0	3 (75.0)	0	
4	0	0	4 (30.7)	
5	0	0	6 (46.1)	
Not available	0	0	2 (15.3)	
Clinical stage, n (%)				0.08
cT1	0	0	0	
cT2	4 (100)	4 (100)	6 (46.1)	
cT3	0	0	7 (53.8)	
cT4	0	0	0	
PPAT* *R*^WO**^ by cut (n)	30.5±16.0 (610)	49.0±30.7 (431)	52.9±72.5 (1751)	<.0001
Mean *R*^*WO*^ tumor fat region	25.38±16.65	34.90±15.72	29.59±14.10	0.158

*PPAT= Periprostatic adipose tissue

**R^*WO*^ = Water-to-oil ratio

Using the mean *R*^WO^ per patient (*n* = 21) based solely on *R*^WO^, the receiver operating characteristic (ROC) curve analysis for identifying high-risk patients yielded an area under the curve (AUC) of 0.61 (SD 0.05). When using initial PSA (iPSA) for this purpose, the AUC improved to 0.84 (SD 0.10). By incorporating *R*^WO^ from chemical shift MRI, the AUC further increased to 0.90, highlighting the potential benefits of combining noninvasive imaging techniques with serum PSA levels for risk stratification (Fig. 1G).

**Fig. 2:**
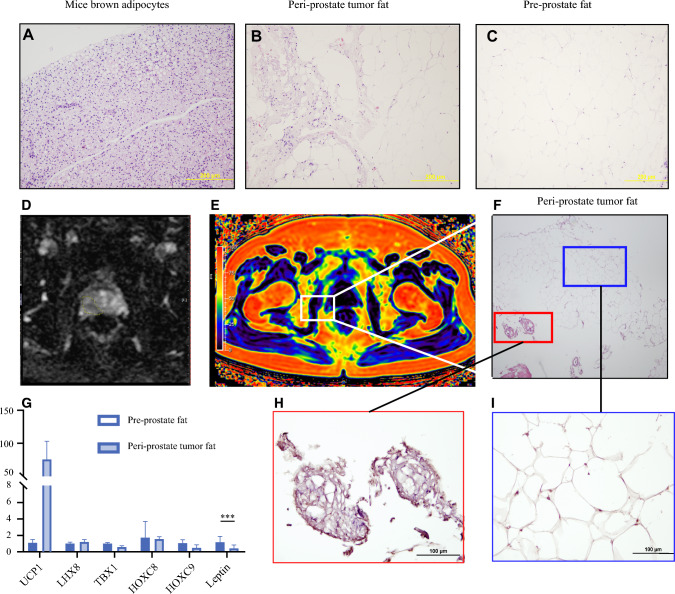
**A**–**C** Hematoxylin and eosin stain of periprostatic fat, (A) mice interscapular BAT as positive control, (B) human adipocytes of periprostate tumor region, (C) human adipocytes of pre-prostate fat distant from tumor, and **D** apparent diffusion coefficient revealing the tumor located at right peripheral zone; **E** chemical shift MRI imaging showing orientation of periprostate tumor fat collected during surgery; **F** hematoxylin and eosin stain (H&E stain) in periprostate fat near tumor showing two different types of adipocyte; **G** qRT-PCR in periprostatic tumor fat and subcutaneous fat; increased expression of BAT-specific markers including UCP1, TBX, and LHX8 in periprostatic tumor fat compared with preprostate fat; increased expression of WAT-specific markers including HOXC8, HOXC9, and Leptin in preprostate fat compared with periprostatic tumor fat; ; **H**–**I** different signal intensities of UCP-1 in IHC reveals the distribution of BAT (red box) and WAT (blue box) within periprostatic fat near tumor region

To elucidate the relationship between distance from the tumor and the browning sequence in periprostate fat, a series of hematoxylin and eosin (H&E) and immunohistochemistry stains were performed on tissue samples near to and distant from the tumor (Fig. 2A,C). We utilized mouse interscapular adipose tissue as a positive control (Fig. 2A) since it contains multilocular lipid droplets and abundant mitochondria, characteristic of BAT, which enhances energy expenditure through thermogenesis, primarily mediated by UCP1.^20^ Our results showed that mouse interscapular adipose tissue expressed abundant UCP1. In the peritumor region, we observed the presence of polygonal adipocytes with multilocular, intracellular lipid droplets (Fig. 2B). Conversely, we found spherical adipocytes containing a large, single lipid droplet (Fig. 2C) in the preprostate fat distant from the tumor region. Prior to sample collection, tumor locations were identified using mpMRI (Fig. 2D), and the *R*^WO^ of periprostatic fat was calculated using chemical shift MRI imaging (Fig. 2E). Immunohistochemistry (IHC) staining for UCP-1 in the periprostate fat revealed both polygonal adipocytes with multilocular, intracellular lipid droplets, suggesting brown adipocytes, and clusters of spherical adipocytes with a single, large lipid droplet, indicative of white adipocytes (Fig. 2F). The varying signal intensities of UCP-1 in immunohistochemistry revealed the distribution of BAT and WAT within the periprostatic fat near the tumor region, correlating with the higher water-to-fat ratio observed in mpMRI imaging.

**Fig. 3:**
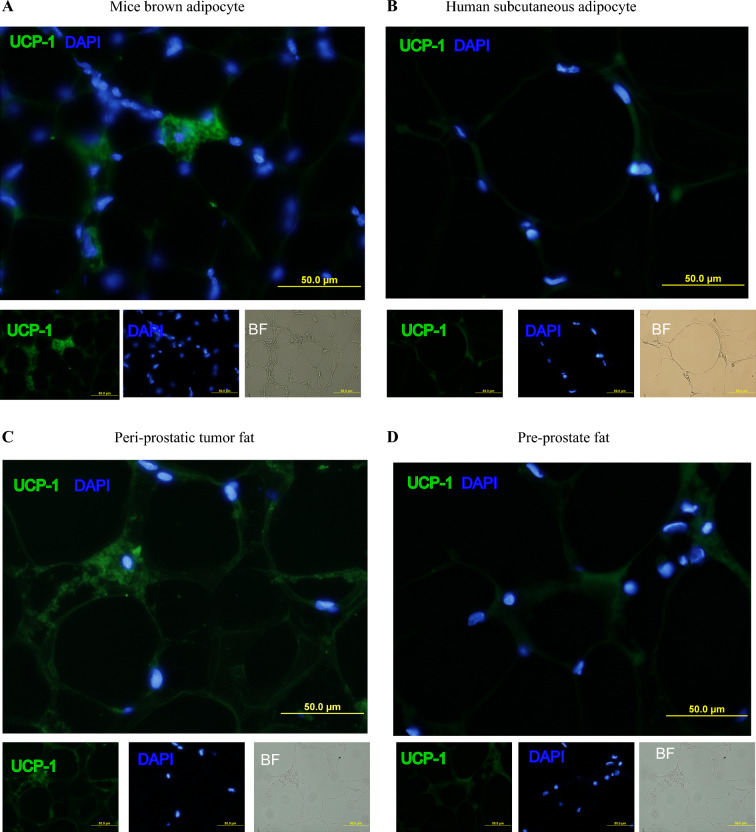
Immunofluorescence staining (IF) with primary antibody UCP-1 Rb mAb 1:50 dilution revealed positive uncoupling protein 1 (UCP-1) signal within periprostate tumor fat (**A**–**D**); (A) interscapular fat containing BAT collected in mice; (**B**–**D**) periprostate fat tissue collected in patient number 6: (B) human subcutaneous fat (400×), (C) periprostate tumor fat (400×), and (D) preprostate fat tissue (400×)

The immunofluorescence (IF) images demonstrated the presence of brown adipocytes with detectable UCP-1 signals within periprostate tumor fat (Fig. 3A,D). The positive control consisted of brown fat from mice, typically located at the back of the neck (Fig. 3A), serving as a reference to confirm the presence of UCP-1 in brown adipocytes. For the negative control, subcutaneous fat from the same patient was used, revealing minimal UCP-1 expression (Fig. 3B). UCP-1 levels were specifically targeted using IF labeling, indicating the presence of brown adipocytes within the periprostate tumor fat (Fig. 3C). A relatively lower expression of UCP-1 was observed in preprostate fat distant from the tumor (Fig. 3D). These images provide evidence of the presence of brown adipocytes, marked by UCP-1 expression, within the periprostate tumor fat. The findings suggest a potential link between brown adipocytes and the tumor microenvironment, indicating their potential role in prostate cancer biology.

**Fig. 4:**
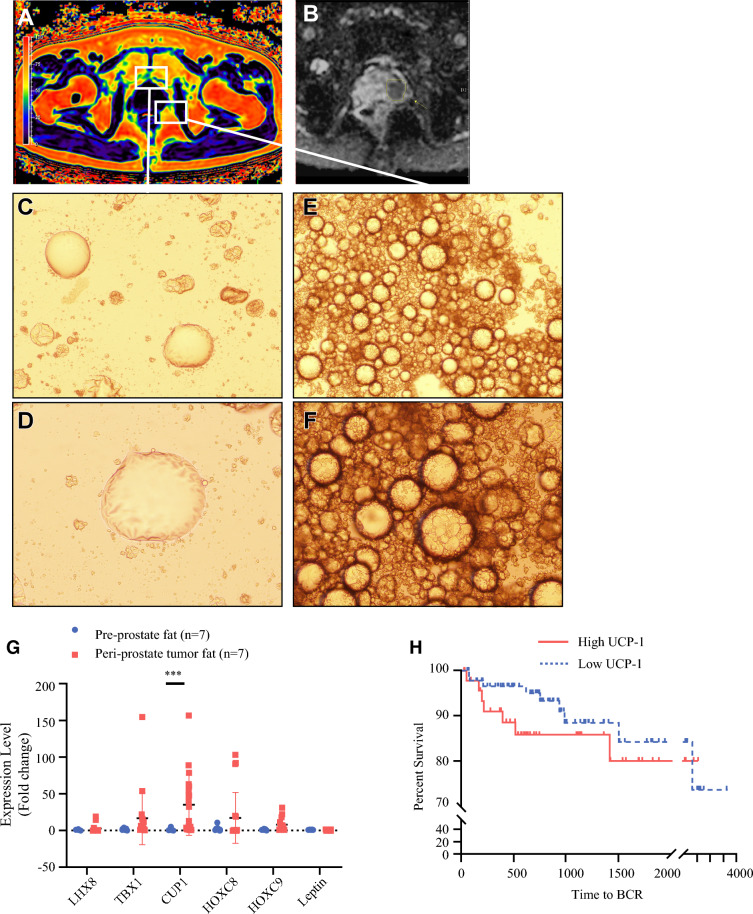
UCP1-positive brown adipocyte within periprostate tumor fat; **A** chemical shift modified DIXON MRI image depicting the orientation of periprostate fat; **B** apparent diffusion coefficient sequence of image showing the location of prostate tumor; **C**–**F** cell culture at 1 week in periprostate fat near tumor and periprostate fat distant from tumor; (C,D) adiopose tissue distant from tumor after 1 week showing cluster of spherical adipocytes containing a large, single lipid droplet suggesting white adipocyte; (E,F) primary culture result of periprostate adipose tissue showing multilocular droplets, spherical and centrally located nuclei suggesting brown adipocyte; **G** qRT-PCR results reveal significantly higher expression of UCP1 within periprostate tumor fat compared with preprostate fat; **H** Kaplan–Meier survival analysis of UCP-1 expression and time to biochemical recurrence in patients with prostate cancer

We utilized chemical shift MRI imaging to calculate the *R*^WO^ of peri-prostatic fat and multiparametric MRI to locate tumors (Fig. 4A,B). After a week of primary culture, adipose tissue from areas distant to the tumor showed characteristics of white adipocytes, with clusters of spherical cells containing a single large lipid droplet (Fig.4C,D). Conversely, adipose tissue near the tumor exhibited features of brown adipocytes, with multilocular droplets and centrally located nuclei (Fig. 4E,F), suggesting a presence of BAT in the periprostate tumor region. Quantitative reverse transcription PCR (qRT-PCR) analysis revealed that BAT markers (UCP-1, TBX1, and LHX8) were significantly more expressed in periprostatic tumor fat compared with preprostate fat (UCP-1 fold change: 35.2 versus 1.30; *p* < 0.001), suggesting a higher presence of UCP-1 expressing BAT near prostate tumors. In contrast, WAT markers (HOXC8, HOXC9, and leptin) showed higher expression in preprostate fat (fold change: 1.01 versus 0.621; *p* < 0.001) (Fig. 4G). Analysis of the open access University of California, Santa Cruz (UCSC) Genomic Online Resource database for the GDC The Cancer Genome Atlas Program (TCGA) Prostate Cancer (PRAD) cohort (*n* = 142) revealed an inverse relationship between UCP-1 expression and time to biochemical recurrence in patients with prostate cancer (hazard ratio, HR: 0.63, 95% confidence intervals, CI: 0.22–1.8, *p* = 0.160) (Fig. 4H).

**Fig. 5:**
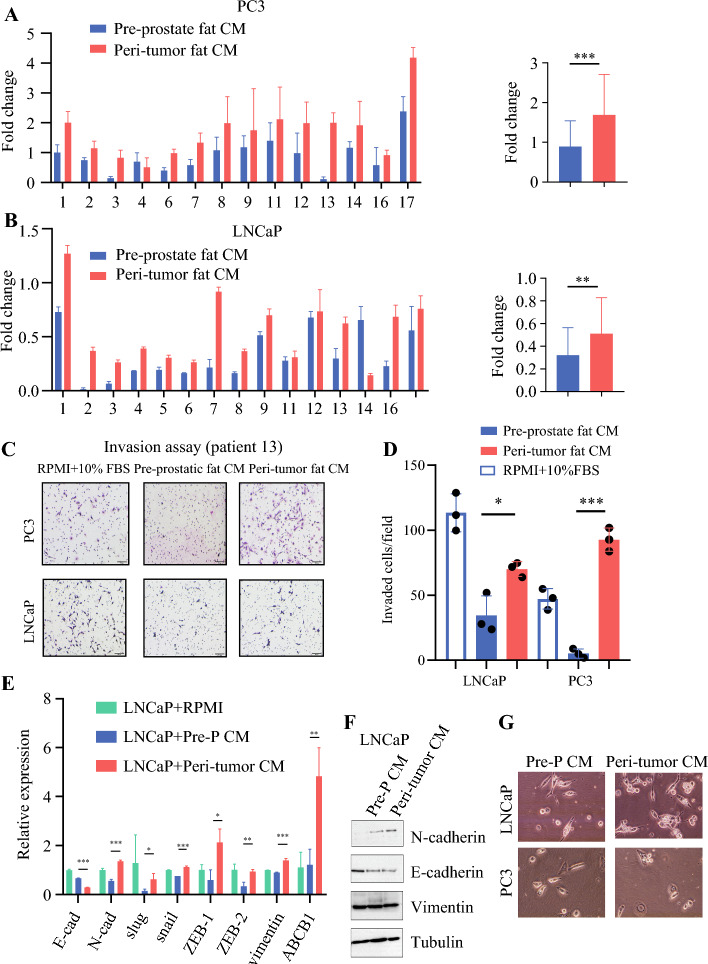
**A**–**D** Transwell invasion assay in different periprostate adipose culture medium groups; (A) representative images of the invasion assay in PC3 cells; PC3 cell invasion through collagen-coated inserts after a 24 h exposure to medium containing supernatant of peritumor adipocyte culture suggesting periprostate tumor fat affects prostate cancer cell invasion potential (*n* = 14); (B) LNCaP cell invasion from collagen-containing inserts after a 24-h exposure to medium containing supernatant of peritumor adipocyte culture (*n* = 15); all signals were normalized to 10.0% FBS (C–D) Representative images of the invasion assay from patient number 13 showing increased cancer cell migration potential attracted by peri-tumor fat culture medium. **E**–**G** Peri-tumor adipocyte co-culture with prostate cancer cell. Periprostate tumor fat affects prostate cancer cell invasion potential is partially contributed by epithelial mesenchymal transition. (E) RT-PCR showing periprostate tumor fat exhibit higher RNA transcription of N-cadherin, Slug, snail, ZEB1,ZEB2, vimentin, and ABCB1 and a lower E-cadherin. (F) Western blots of LNCaP cancer cells 72 hours after co-culture with human peri-prostatic adipocyte cultured after 1 week showing decreased E-cadherin and increased N-cadherin. (G) Cell morphology of LNCaP and PC3 co-cultured with pre-prostate fat conditioned medium and periprostate fat conditioned medium for 72 hours.

In the 24-h Transwell assay, LNCaP cells showed a fold change in migration from 0.33 (preprostate fat medium) to 0.55 (periprostate tumor fat medium) (*p* < 0.0003; 95% CI: 0.10–0.33). PC3 cells had a fold change from 0.89 (preprostate fat medium) to 1.69 (periprostate tumor fat medium) (*p* < 0.0001; 95% CI: 0.43–1.17; Fig. 5A,D). The conditioned medium from periprostatic tumor fat significantly increased the migration of these cells compared with medium from distant fat (Fig. 5A,B). Using qRT-PCR, we observed a decrease in E-cadherin and an increase in mesenchymal markers such as Slug, N-cadherin, vimentin, ZEB1, and ZEB2 in LNCaP cells treated with periprostatic tumor fat medium, indicating epithelial–mesenchymal transition (Fig. 5E,F). A 24-ho coculture of PC3 and LNCaP cells with human periprostatic tumor fat led to mesenchymal morphological changes in the cancer cells (Fig. 5G), suggesting the secretome of periprostatic fat cells enhances migratory and invasive capabilities in prostate cancer cells.

## Discussion

Several studies have previously investigated the relationship between PPAT area, volume, ratio (PPAT volume/prostate volume), density, or thickness and PCa clinical features, with controversial results. Rather than using chemical shift imaging, Shahait et al. characterized T1-MRI-derived radiomic parameters of PPAT associated with clinically significant PCa in a cohort of men who underwent robot-assisted prostatectomy.^21^ As machine deep learning has advanced, automatic and accurate segmentation of prostate and periprostatic fat has been made possible.^22^ More accurate and precise evaluation on not only tumors but also periprostatic fat is evolving.^23^ The appeal of MR imaging is that it has the potential to further distinguish brown adipose tissue and white adipose tissue on the basis of its physiologic factors. Previous study had shown that fat-signal fractions and T2 values jointly derived from chemical shift water-fat MRI are lower in BAT than in WAT likely because of differences in cellular structures, triglyceride content, and vascularization.^24^ MRI techniques focusing on adipocyte-related aspects of prostate cancer have the potential to impact diagnosis, risk stratification, and treatment planning. The integration of adipocyte-specific information obtained from MRI can improve the accuracy of prostate cancer detection and characterization.

Being a single center pilot study, our result demonstrates for the first time that there is a significantly higher PPAT water-to-oil ratio (*R*^WO^) compared with the low-risk group (mean 52.12 versus 30.48; 95% CI: 16.32–26.94; *p* < 0.0001), compatible with the immunofluorescence staining and signal intensities of UCP-1 in immunohistochemistry showing the presence of BAT within PPAT collected in localized patients with prostate cancer. In addition, they demonstrate that combining noninvasive chemical shift MRI imaging with serum PSA holds promising prediction value of patients with high-risk prostate cancer. The MRI-based classification system, focusing on the periprostatic adipose tissue water–fat ratio, achieved an AUC of 0.61, indicating its ability to distinguish clinically significant disease from low-risk disease. Incorporating serum PSA into this system improved its performance, increasing the AUC to 0.90, thereby demonstrating a stronger discriminatory power of this noninvasive imaging technique when used alongside serum PSA.

The relatively small sample size limits the ability to draw definitive conclusions; in addition, it is important to note the uneven distribution of the patients across risk groups, with a predominance of high-risk cases. One of the limitations is the lack of clinical PPAT specimens among metastatic group; thus, the clinical relationship between PPAT and metastatic disease remain largely elusive. Moreover, healthy individuals are not included in the study since it would not be possible to define the periprostate tumor area. MRI scans were performed on 1.5-T Siemens MR units; whether the technique could extend to other external validation cohorts using 3-T MR units requires further collaboration involving multiple medical centers. As for clinical relevance, our results demonstrate in the real-world that this method may help in identifying patients with aggressive disease who could benefit from targeted therapies aimed at disrupting adipocyte–tumor cell interactions.

Future research can proceed by increasing the data volume and follow-up time. Further follow-up on the patients’ time to biochemical recurrence is also required for validating its role in assessing the oncological outcomes. Second, it can proceed by experimenting with more data preprocessing and augmentation methods. By applying different degrees of rotation, scaling, and flipping to the images, it may increase data diversity and model robustness. Third, different deep learning model structures and methods can be explored. For instance, utilizing different convolutional neural network (CNN) architectures or trying other types of deep learning methods, such as recurrent neural networks (RNNs), to identify models better suited to addressing the prostate cancer risk classification. Fourth, interpretable models could be developed. In the medical field, a model’s interpretability is crucial for gaining the trust of doctors and patients. Therefore, researching how to enhance the interpretability of deep learning models to better understand and explain classification results will have significant practical value. This knowledge may pave the way for developing novel therapeutic strategies that specifically target the adipocyte–tumor cell crosstalk. Our results confirm the presence of BAT within the tumor microenvironment within PPAT, which affects prostate cancer invasion.

## Conclusions

Our results provide real-world evidence that there is a difference in the fat component between periprostate fat near and distant from tumor region, which correlates to the browning of white adipocyte as to be detected within chemical shift series of MRI. Chemical shift imaging allows researchers to obtain detailed information about the presence, distribution, and metabolic properties of adipocytes in the prostate tumor microenvironment. This knowledge has the potential to enhance our understanding of prostate cancer biology, improve diagnostic accuracy, and guide the development of targeted therapies.

## Supplementary Information

Below is the link to the electronic supplementary material.

Supplementary file1 (DOCX 16 kb)

Supplementary file2

Supplementary file3
