# Supplementary material for: Integrating Magnetic Resonance Chemical Shift Imaging for Localized Prostate Cancer Risk Stratification on the Basis of the Impact of Periprostatic Brown Adipocytes Within Tumor Microenvironment
Source: Ann Surg Oncol. 2025 May 28;32(9):6962–73. doi: 10.1245/s10434-025-17512-5 (PMC12317911; doi:10.1245/s10434-025-17512-5)
Supplement: Supplementary file 1 — Supplementary file1 (DOCX 16 kb) [file 10434_2025_17512_MOESM1_ESM.docx]

**Supplementary Material**

Supplementary Figure 1:

Supplementary Fig. 1A: MRI analysis of periprostatic fat, utilizing water-only signal (WOS) and fat-only signal (FOS). 1B: Image of Water Only Matrix (IWO), representing WOS in a 2D slice from MRI data. 1C: Image of Fat Only Matrix (IFO), showing FOS in the selected MRI slice.1D: Image of the water-to-oil ratio (IRWO), derived from WOS and FOS matrices. 1E: Image of Brown Fat of Periprostatic Fat Ratio (IBPRWO), highlighting brown fat in periprostatic fat using conversion and gamma correction methods. 1F: Heat map overlay on eT2w image showing regions and water-to-oil ratio of brown fat in the periprostatic fat. Brown fat is identified within the green contour and outside the red contour. (Note: Conversion from original data to visual representation involved adjusting intensity levels from 4096 to 256 and employing specific equations [Eq. X and Eq. Y] for calculation.)

Supplementary Figure 2: Distribution of signals of water-fat ratio (*R^WO^*) threshold setting1% of peri-prostate adipose tissue in chemical shift MRI imaging incorporated in MRI T2 series. Relatively concentrated signal within peri-prostate tumor region in clinical significant risk group
