# Supplementary figures and images for: Integrating Magnetic Resonance Chemical Shift Imaging for Localized Prostate Cancer Risk Stratification on the Basis of the Impact of Periprostatic Brown Adipocytes Within Tumor Microenvironment
Source: Ann Surg Oncol. 2025 May 28;32(9):6962–73. doi: 10.1245/s10434-025-17512-5 (PMC12317911; doi:10.1245/s10434-025-17512-5)

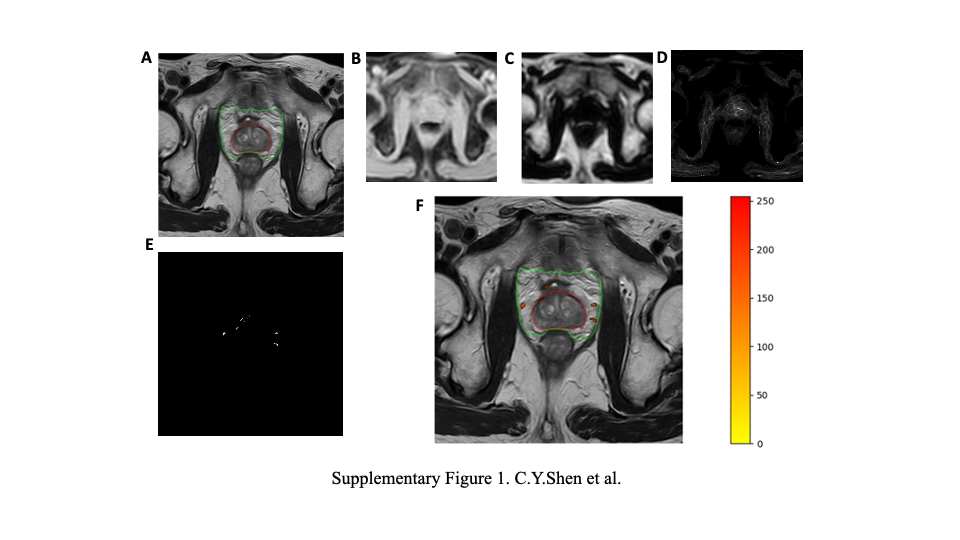

Supplement: Supplementary file 2 — Supplementary file2 [file 10434_2025_17512_MOESM2_ESM.tiff]

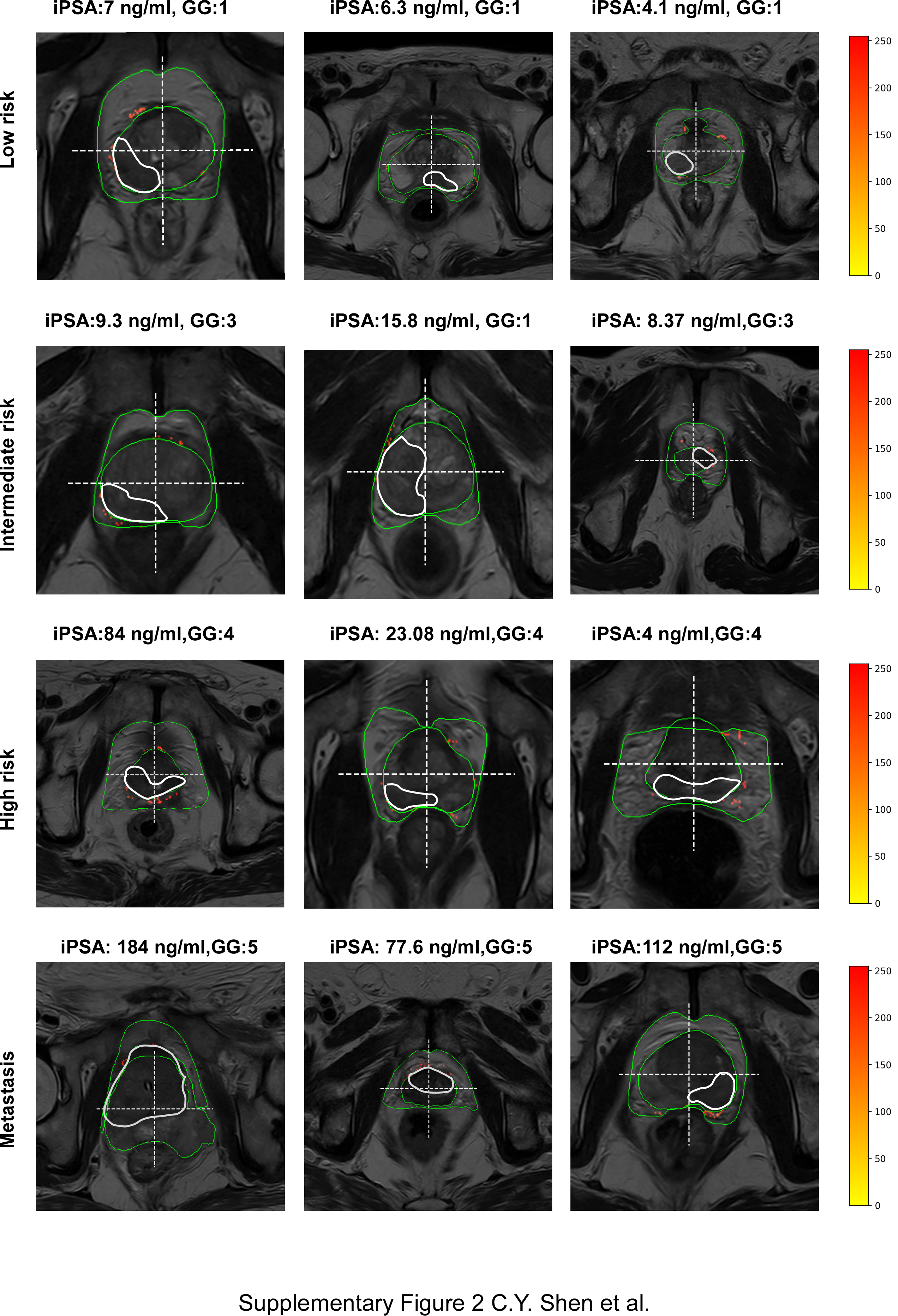

Supplement: Supplementary file 3 — Supplementary file3 [file 10434_2025_17512_MOESM3_ESM.tif]
